# Association between serum vitamin D levels and visceral adipose tissue among adolescents: a cross-sectional observational study in NHANES 2011–2015

**DOI:** 10.1186/s12887-022-03688-2

**Published:** 2022-11-04

**Authors:** Yan-feng Li, Xiao Zheng, Wen-lan Gao, Feng Tao, Yi Chen

**Affiliations:** 1 Department of Rehabilitation, Shanghai Municipal Hospital of Traditional Chinese Medicine, Shanghai University of Traditional Chinese Medicine, Shanghai, 200071 China; 2 Department of Endocrinology, Shanghai Municipal Hospital of Traditional Chinese Medicine, Shanghai University of Traditional Chinese Medicine, Shanghai, 200071 China

**Keywords:** Vitamin D, Visceral adipose tissue, Adolescent, NHANES

## Abstract

**Background:**

In recent years, obesity and vitamin D deficiency are more prevalent among adolescents. Improving our knowledge of the link between vitamin D and visceral adipose tissue (VAT) is essential for the health of adolescents. This study aimed to examine the connection between serum vitamin D levels and VAT mass among adolescents participating in the United States.

**Methods:**

This is a cross-sectional study that used data from the 2011 to 2015 National Health and Nutrition Examination Survey (NHANES). The connection between serum vitamin D levels and VAT was investigated using weighted multiple linear regression models. Potential nonlinear relationships were explored using smooth curve fitting.

**Results:**

The analysis included 3171 adolescents aged 12–19 years. Vitamin D levels were shown to be inversely linked with VAT in the full-adjusted model (β = − 0.34, 95% CI: − 0.49 to − 0.19). When stratified analyses by gender, this negative relationship persisted in the girls’ group (β = − 0.39, 95% CI: − 0.60 to − 0.19), but not in the boys’ group (β = − 0.06, 95% CI: − 0.25 to 0.13). When stratified analysis by race, this negative relationship persisted in the Mexican American group (β = − 0.61, 95% CI: − 1.03 to − 0.19), and the non-Hispanic White group (β = − 0.27, 95% CI: − 0.54 to − 0.01), but not in the other groups.

**Conclusions:**

Our findings confirmed that serum vitamin D levels negatively correlated with VAT among adolescents in the United State, especially in girls, the Mexican American and non-Hispanic White. Further research is needed to determine whether increasing serum vitamin D levels decrease VAT among adolescents.

**Supplementary Information:**

The online version contains supplementary material available at 10.1186/s12887-022-03688-2.

## Background

Obesity is a multifactorial state of chronic excess fat accumulation linked to serious health risks [1]. Obesity leads to insulin resistance, diabetes, and hypertension, greatly increasing cardiovascular disease (CVD) morbidity and mortality [2, 3]. It has been demonstrated that abdominal fat deposition is superior to whole-body fat in predicting coronary artery risk [4]. Furthermore, the Framingham heart study confirmed that stronger association of visceral adipose tissue (VAT) with some common cardiovascular and metabolic risk variables than subcutaneous adipose tissue (SAT) [5].

Vitamin D’s primary function is to keep calcium metabolism in check and preserve bone health [6]. In addition, serum vitamin D has a critical role in many prevalent chronic conditions, including CVD, metabolic syndrome, obesity, insulin resistance, prediabetes and type 2 diabetes [7–9]. According to a meta-analysis, vitamin D is inversely connected to cardiovascular risk [10].

Numerous studies have long identified a relationship between vitamin D deficiency and obesity [11–13]. Vitamin D is a vital fat-soluble vitamin that can be stored in adipose tissue. There is a possibility that a lower level of serum vitamin D may be caused by fat sequestration [14] or volumetric dilution [15] in obesity. Because VAT plays an important function in the cardiovascular system [5], some scholars have explored the link between serum vitamin D levels and visceral fat. Two large-scale epidemiological surveys have reported a negative link between VAT thickness and serum vitamin D concentration in adults [16, 17]. However, less research has been done on adolescents. Two medium-sized, regionally limited studies suggested that vitamin D is inversely associated with VAT in adolescents [18, 19]. This study explored the correlation between the two in American adolescents using a large, multi-ethnic population database sample.

## Methods

### Study population

**Fig. 1:**
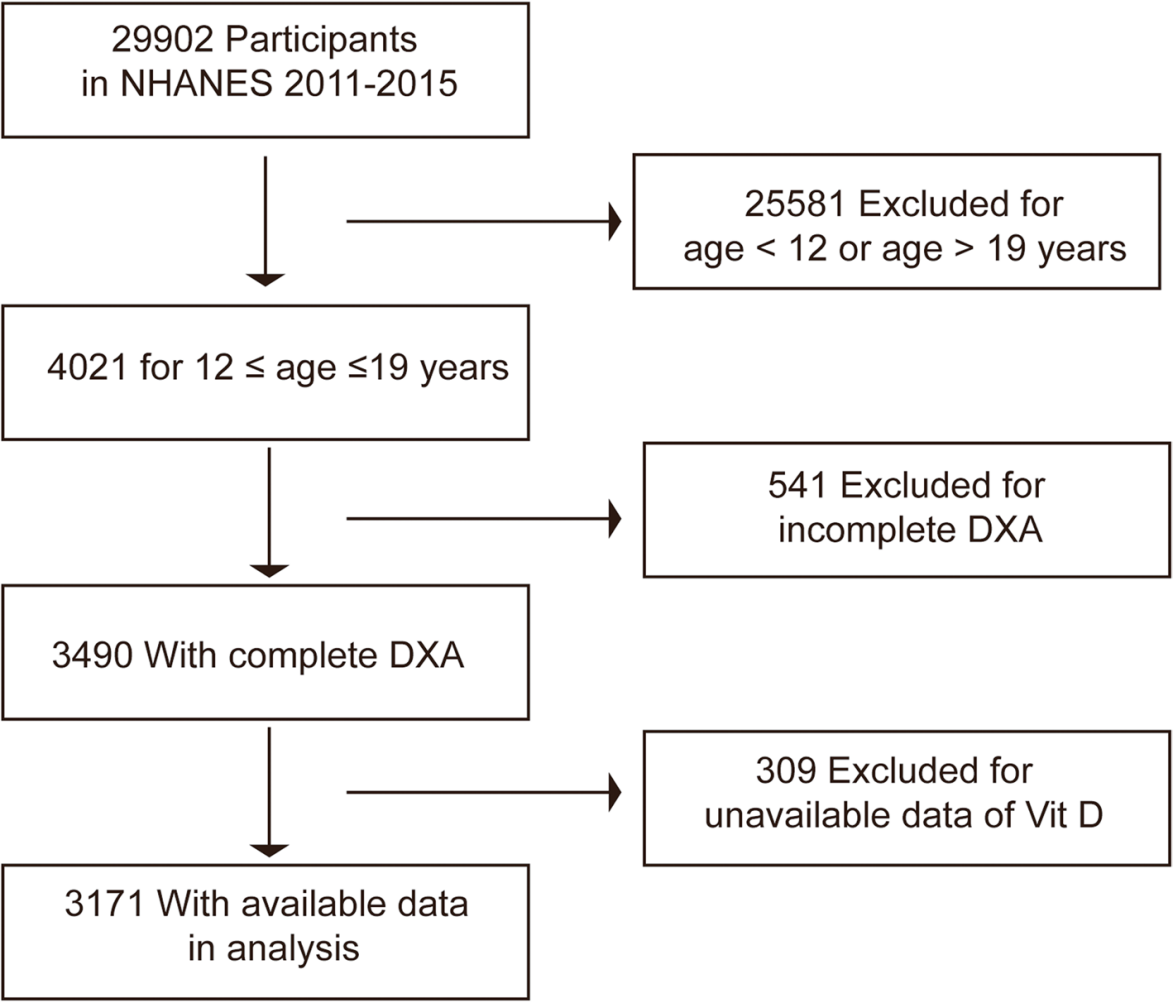
A flowchart showing the selection of study participants. DXA, dual-energy X-ray absorptiometry

The National Health and Nutrition Examination Survey (NHANES) is a nationally representative survey that gathers health screening information from the U.S. non-institutionalized population [20]. The nationwide study is conducted every 2 years, continuously. The data used in this study are from three consecutive NHANES cycles from 2011 to 2016. As shown in Fig. 1, this study included 3171 subjects who had complete information on serum vitamin D levels and VAT mass aged between 12 and 19. The NHANES procedure was approved by the Institutional Review Committee of the National Center for Health Statistics, and all participants or their agents (< 18 years old) completed the informed permission forms [21, 22].

### Variables

The dependent variable was VAT mass in this study, and all included participants were given whole-body scans using dual-energy X-ray absorptiometry (DXA). In the 2011–2016 cycles, VAT mass was assessed by qualified and certified radiation technologists using the DXA scans obtained from the Hologic Discovery model A densitometer (Hologic, Inc., Bedford, Massachusetts, software version Apex 3.2).

The independent variable in this study was serum vitamin D levels obtained by the radioimmunoassay kit (DiaSorin, Stillwater, Minnesota, USA) during the 2011–2016 cycles. In addition, serum vitamin D levels were split into three main categories: deficiency (< 50 nmol/L), insufficient (50–75 nmol/L), and sufficient (≥75 nmol/L) [23].

According to previous studies [18, 24], age, sex, race, sedentary activity, income-to-poverty ratio, time of detection, and BMI category were identified as potential confounding factors. The race was classified as Mexican American, other Hispanic, non-Hispanic White, non-Hispanic Black, and other races, including Alaska Natives or American Indians, Native Hawaiians or other Pacific Islanders, and multiracial individuals. The season is an important factor in vitamin D status, so this study included the time of detection, which is defined as a dichotomous variable based on the time of detection: November 1 through April 30 and May 1 through October 31 [25]. BMI category is for children and adolescents aged 2 to 19 years. Cutoff criteria are based on the Centers for Disease Control and Prevention’s sex-specific 2000 BMI-for-age growth charts for the United States. There are four codes, 1: Underweight (BMI < 5th percentile);2: Normal weight (BMI 5th to <85th percentiles); 3: Overweight (BMI 85th to <95th percentiles); 4: Obese (BMI ≥ 95th percentile). In addition, the level of income was measured using the poverty income ratio (ratio of family income to poverty threshold) and was classified into three categories: < 1.3, 1.3–1.8, and > 1.8. All details about serum vitamin D levels, VAT mass, and other variables are recorded at www.cdc.gov/nchs/nhanes/.

### Statistical analysis

According to NHANES’s recommendation, all analyses used the sample weights derived from designs based on stratified, multistage probabilistic sampling. For categorical and continuous variables, *P* values were obtained using the weighted chi-square test and the linear regression model, respectively. And two-sided *P*-value less than 0.05 was deemed statistically significant. The weighted multiple linear regression models adjusted for different confounders were performed to explore whether serum vitamin D levels were independently related to VAT mass. Model 1 did not include any covariates; Model 2 adjusted for age, gender and race; Model 3 further adjusted the covariates, which satisfy one of the following conditions: (1) the regression coefficients *P*-value for covariates on VAT mass < 0.10, or (2) the incorporation of covariates in the model causes a more than 10% change in regression coefficients. Generalized additive model and smooth curve fitting (penalized spline method) were tried to explain the potential nonlinear relationships after adjusting the same covariates. In addition, subgroup analysis and interaction analysis were conducted to assess the impact of gender or race on the outcome. All data analyses were performed using EmpowerStats and the R software (version 3.5.3).

## Results

This study included 3171 eligible adolescents with complete data on serum vitamin D and VAT. The missing data of covariates are shown in supplementary Table 1. For categorical variables, missing data were included as a ‘not recorded’ category. For continuous variables, missing data were encoded by the weighted mean. After the missing data were filled, the relationships between covariates and VAT mass were shown in supplementary Table 2. Table 1 shows weighted characteristics divided into three categories according to serum vitamin D levels: deficiency, insufficiency, and sufficiency. Participants in different groups differed significantly in several characteristics. Compared to the deficient group, participants in the insufficiency and sufficiency groups had higher income, but less sedentary activity time, lower waist circumference, VAT mass, and less obesity.

**Table 1:** Weighted characteristics of study population based on serum vitamin D levels

Characteristic	Deficiency1272 (< 50 nmol/L)	Insufficiency1425 (> = 50, < 75 nmol/L)	Sufficiency474 (> = 75 nmol/L)	*P* value
Age, mean ± SD (years)	15.55 ± 2.24	15.28 ± 2.22	15.45 ± 2.22	0.0143
Gender (%)				< 0.0001
Boys	47.47	58.08	49.62	
Girls	52.53	41.92	50.38	
Race (%)				< 0.0001
Mexican American	25.59	14.71	4.00	
Other Hispanic	10.12	8.43	2.83	
Non-Hispanic White	21.67	58.41	85.28	
Non-Hispanic Black	31.97	9.30	2.24	
Other Race	10.65	9.15	5.65	
Ratio of family income to poverty (%)				< 0.0001
< 1.3	42.16	27.21	18.84	
1.3–1.8	13	11.13	7	
> 1.8	36.47	55.29	70.41	
Not recorded	8.37	6.37	3.75	
Time of detection (%)				< 0.0001
November 1 through April 30	59.22	48.06	26.83	
May 1 through October 31	40.78	51.94	73.17	
Sedentary activity, mean ± SD, (minutes)	513.29 ± 177.52	488.65 ± 157.54	465.38 ± 156.75	< 0.0001
Waist circumference, mean ± SD, (cm)	85.80 ± 17.29	82.64 ± 15.31	79.17 ± 11.69	< 0.0001
BMI				< 0.0001
Underweight	3.85	3.83	2.75	
Normal weight	47.98	55.78	71.52	
Overweight	17.51	18.4	13.1	
Obese	30.03	21.17	11.26	
Not recorded	0.63	0.83	1.38	
Serum vitamin D, mean ± SD (nmol/L)	38.73 ± 8.57	61.86 ± 7.01	92.41 ± 17.63	< 0.0001
Visceral adipose tissue mass (mg)	267.48 ± 166.11	244.06 ± 133.50	208.22 ± 108.58	< 0.0001

Mean ± S.D. for continuous variables: *P* value was calculated by weighted linear regression model

% for categorical variables: *P* value was calculated by weighted chi-square test

*Abbreviation*: *BMI* Body mass index

Table 2 shows the relation between serum vitamin D levels and VAT mass for the three linear regression models. The relation between serum vitamin D levels and VAT mass was negatively correlated in the unadjusted model (β = − 1.23, 95% CI: − 1.44 to − 1.02). And this negative correlation was still maintained in the fully adjusted model (β = − 0.34, 95% CI: − 0.49 to − 0.19). In addition, the trend remained significant among the different serum vitamin D level groups (P for trend < 0.001).

**Table 2:** Association between serum vitamin D levels (nmol/L) and VAT mass (mg)

	Model 1		Model 2		Model 3	
	β (95% CI)	*P* value	β (95% CI)	*P* value	β (95% CI)	*P* value
Serum vitamin D levels (nmol/L)	−1.23 (− 1.44, − 1.02)	< 0.0001	− 1.63 (− 1.86, − 1.39)	< 0.0001	−0.34 (− 0.49, − 0.19)	< 0.0001
Serum vitamin D (quartile)						
Deficiency	Reference		Reference		Reference	
Insufficiency	−23.42(− 34.94,-11.90)	< 0.0001	−37.93(− 50.06,-25.81)	< 0.0001	−8.30 (− 15.42, − 1.18)	0.0224
Sufficiency	−59.26(− 72.50,-46.02)	< 0.0001	−78.22(− 92.95,-63.48)	< 0.0001	− 14.70(− 23.62,-5.78)	0.0012
P trend	< 0.001		< 0.001		0.001	

Model 1 adjusted for: None

Model 2 adjusted for age, race, and gender

Model 3 adjusted for age, race, gender, sedentary activity, ratio of family income to poverty, time of detection, BMI, and waist circumference

Further subgroup analyses by gender and race were performed (Table 3). When stratified analyses by gender, the relation between serum vitamin D levels and VAT mass was negatively correlated in model 1 and model 2. Adjusted all the covariates, this negative relationship persisted in the girls’ group (β = − 0.39, 95% CI: − 0.60 to − 0.19), but not in the boys’ group (β = − 0.06, 95% CI: − 0.25 to 0.13), and the test for interactions was significant (p interaction = 0.0191). When stratified analyses by race, this negative relationship still appeared in model 1 and model 2. Adjusted all the covariates, this negative relationship persisted in the Mexican American group (β = − 0.61, 95% CI: − 1.03 to − 0.19) and the non-Hispanic White group (β = − 0.27, 95% CI: − 0.54 to − 0.01), but not in the other groups.

**Table 3:** Association between serum vitamin D levels (nmol/L) and VAT mass (mg) stratified by race and gender

	Model 1		Model 2		Model 3		
	β (95% CI)	*P* value	β (95% CI)	*P* value	β (95% CI)	*P* value	P interaction
Stratified by race							0.4770
Mexican American	−2.57 (−3.28, − 1.86)	< 0.0001	− 2.60 (− 3.32, − 1.88)	< 0.0001	− 0.61 (− 1.03, − 0.19)	0.0046	
Other Hispanic	−1.22 (− 2.04, − 0.41)	0.0035	−1.20 (− 2.01, − 0.38)	0.0042	− 0.33 (− 0.83, 0.17)	0.1924	
Non-Hispanic White	−1.78 (− 2.21, − 1.35)	< 0.0001	−1.81 (− 2.24, − 1.38)	< 0.0001	− 0.27 (− 0.54, − 0.01)	0.0395	
Non-Hispanic Black	− 0.86 (− 1.36, − 0.35)	0.0009	−0.95 (− 1.45, − 0.45)	0.0002	−0.02 (− 0.36, 0.32)	0.9182	
Other Race	−0.57 (− 1.09, − 0.04)	0.0339	−0.61 (− 1.13, − 0.09)	0.0219	−0.22 (− 0.55, 0.12)	0.2032	
Stratified by gender							0.0191
Boys	−0.87 (− 1.16, − 0.58)	< 0.0001	− 1.15 (− 1.46, − 0.84)	< 0.0001	−0.06 (− 0.25, 0.13)	0.5423	
Girls	−1.43 (− 1.74, − 1.12)	< 0.0001	−2.02 (− 2.38, − 1.66)	< 0.0001	−0.39 (− 0.60, − 0.19)	0.0002	

Model 1 adjusted for: None

Model 2 adjusted for age, race, and gender

Model 3 adjusted for age, race, gender, sedentary activity, ratio of family income to poverty, time of detection, BMI, and waist circumference

In the subgroup analysis stratified, the model is not adjusted for the stratification variable itself

**Fig. 2:**
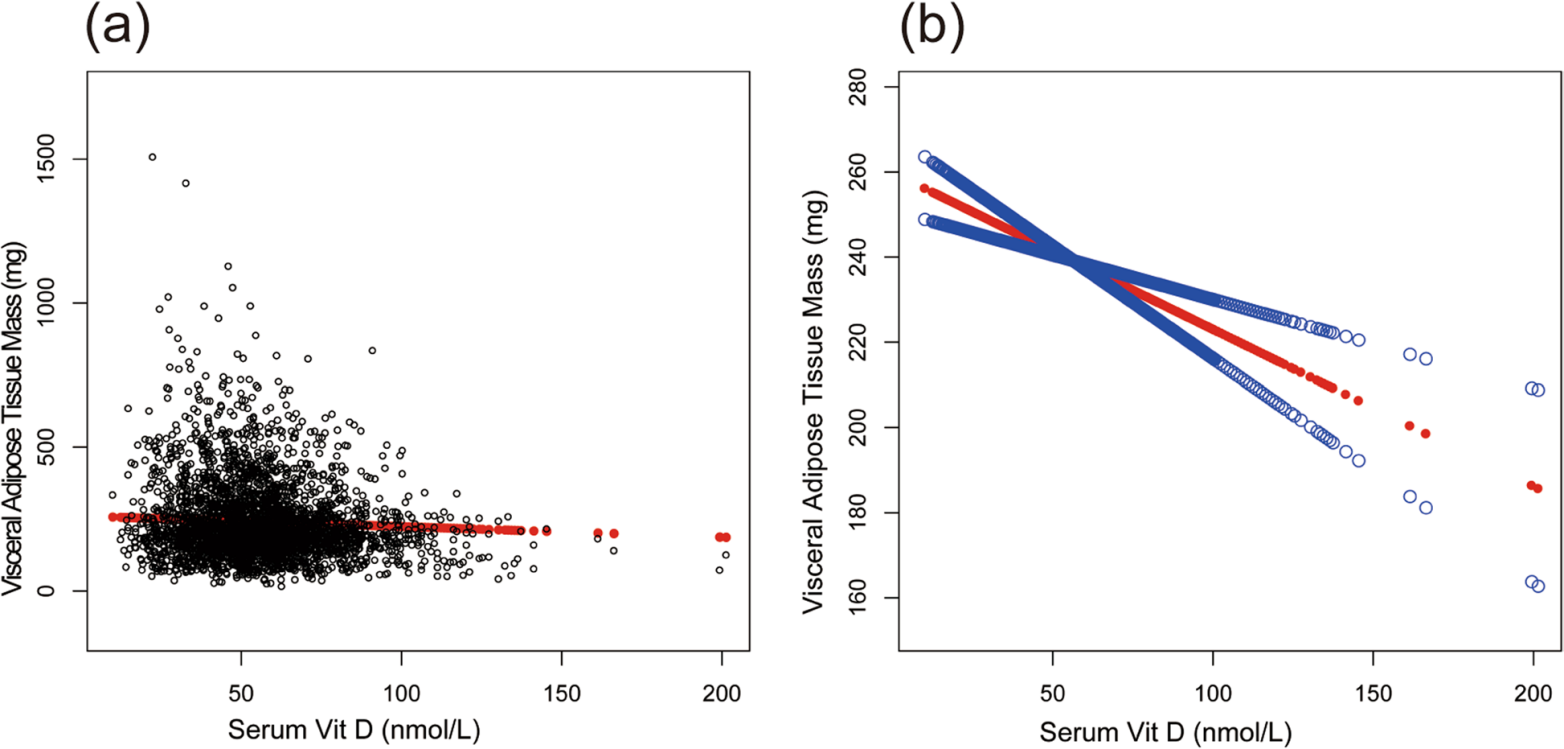
The association between serum Vit D and visceral adipose tissue mass. **a** Each black point represents a sample. **b** Solid red line represents the smooth curve fit between variables. Blue lines represent the 95% confidence interval of the slope of the fitting line. Adjusted for age, race, gender, sedentary activity, ratio of family income to poverty, time of detection, BMI, and waist circumference

**Fig. 3:**
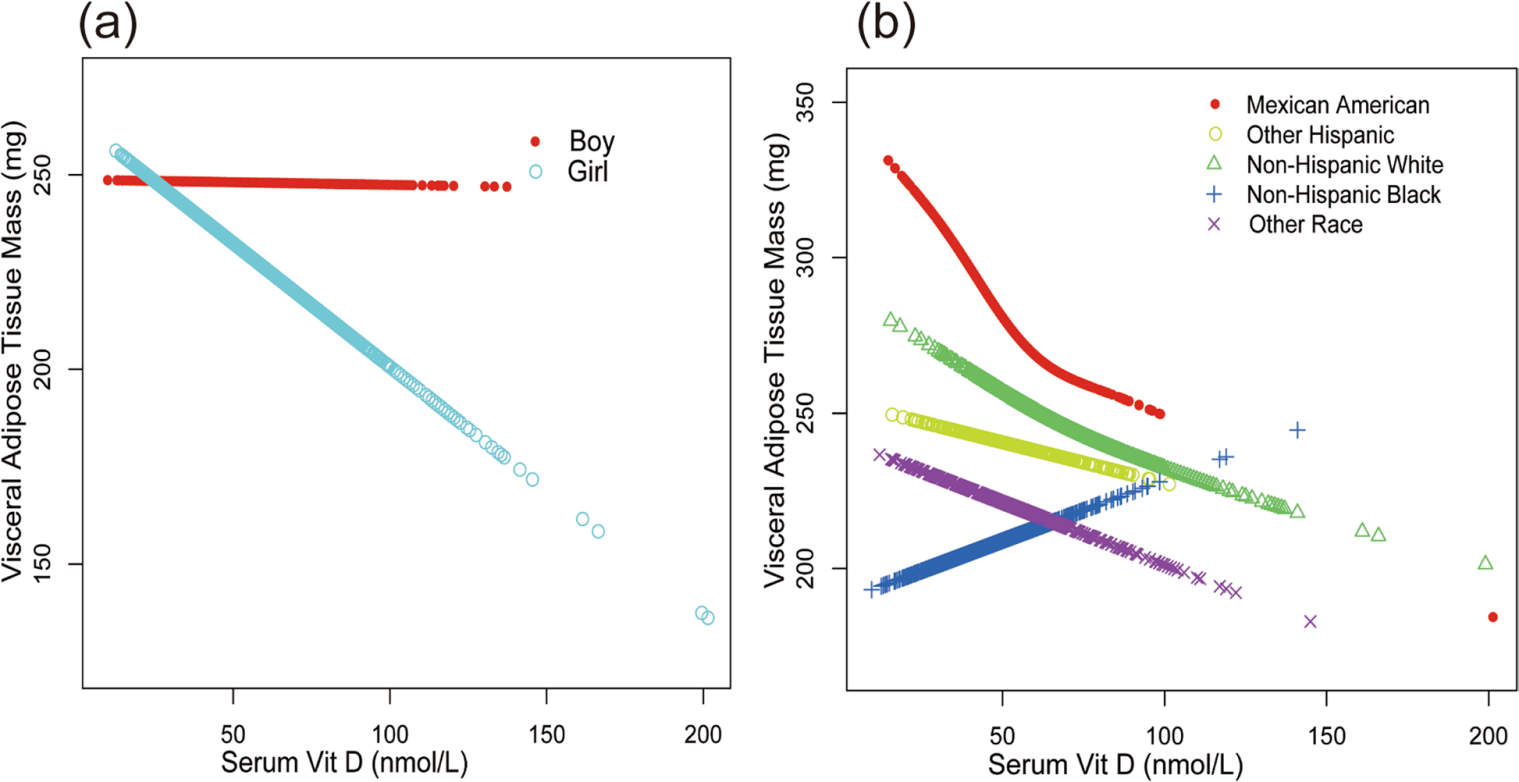
The association between serum Vit D and visceral adipose tissue mass stratified by gender (**a**) and race (**b**). Adjusted for age, race, gender, sedentary activity, ratio of family income to poverty, time of detection, BMI, and waist circumference

In Fig. 2, the smooth curve fitting illustrated the VAT mass decreased linearly with the increase of serum vitamin D levels, which further confirmed this inverse association. In Fig. 3, we intuitively observed that in the subgroup analysis, except for the Non-Hispanic black group, the VAT mass decreased linearly with the increase of serum vitamin D levels.

## Discussion

This study aimed to explore whether there is an independent link between serum vitamin D concentrations and VAT in adolescents. Our study analyzed their association using NHANES data, and found that VAT mass decreased with the increase of serum vitamin D levels in American adolescents.

Evidence suggests that the metabolism, storage, and function of vitamin D can affect obesity and be affected by obesity. Some studies have shown that obesity may lead to vitamin D insufficiency [23, 24], which may be because people of obese have more VAT, which can store more vitamin D [14]. This may have resulted in less vitamin D being released into the bloodstream, so these people had lower serum vitamin D levels. Reduced outdoor exercise and increased clothing covering the skin also reduced the photochemical subcutaneous production of vitamin D in overweight people [26]. Meanwhile, vitamin D deficiency may lead to obesity in adults and children [27, 28]. There are shreds of evidence that 1,25-(OH)_2_D may induce lipogenesis and inhibits lipolysis [29, 30], which may be due to the flow of calcium into adipocytes by increasing parathyroid hormone synthesis [31, 32].

Fat distribution affects obesity outcomes, and the increase in visceral fat can affect people’s health [5, 33, 34]. Most studies on the link between VAT and vitamin D levels come from adult data. The Framingham Heart study [16] reported that people with high VAT were more deficient in vitamin D than those with low VAT. Other studies had come to a similar conclusion that VAT was inversely associated with serum vitamin D levels [17, 35]. However, there is currently little research on VAT and vitamin D in adolescents. In a cross-sectional cohort(*n* = 237), the researchers discovered an independent negative connection between VAT and serum 25(OH) D levels in individuals aged 8-18y in Pittsburgh [18]. In another medium-sized study (*n* = 559), the concentration of 25(OH)D was shown to be substantially adversely linked with visceral fat in adolescents aged 14-18y in Atlanta [19]. The results of this study are the same as the above results, and the larger sample size (*n* = 3171) makes this analysis have stronger statistical power. And further subgroup analysis found the different effects of gender and race.

It is well recognized that obesity levels and body fat distribution vary across races and ethnicities [36–39]. For the same BMI, Caucasian children are more obese than African-American children [37]. Additionally, abdominal adipose tissue is distributed differently by ethnicity: a survey found that Caucasian children have higher VAT and lower SAT than African-American children [18]. The same study also showed that the negative relationship between serum vitamin D and VAT existed only in whites, but the same negative relationship was found in blacks after excluding abnormal values [18]. However, this study did not find a negative relationship between serum vitamin D and VAT in blacks. Although ethnic differences in the extent of vitamin D accumulation in adipose tissue may explain these discrepancies, they are more likely owing to confounding effects produced by race variances in skin color [40]. Race-related visceral adiposity differences may also be the reason for the different associations between obesity and vitamin D among different ethnic groups [36, 41]. Therefore, further investigation research is needed to observe the relation of serum vitamin D with obesity in childhood of various races.

Previous research suggested that the relation between serum vitamin D level and visceral fat differ between men and women. A study using bioelectrical impedance analysis showed that less visceral fat area reduced the incidence of vitamin D deficiency in premenopausal women and men [42]. A Brazilian study of 190 pregnant women found no link between vitamin D and visceral fat on ultrasonography [43]. However, another study using CT observed an inverse connection between 25(OH)D and visceral fat area in healthy young females aged 16–22 years [44]. This study found that in sex-stratified analysis, the negative connection between serum vitamin D levels and VAT mass only exists in girls. The inconsistency of vitamin D-VAT association in these results may be explained by the lack of similarity in assay methods, individual growth and developmental stages among the different research.

There are some limitations to this study. Firstly, because this was a cross-sectional investigation, the causal relationship between serum vitamin D levels and VAT could not be established. Secondly, our research is conducted among teenagers, so it cannot be extended to other groups. However, our research has some strengths. The data come from a representative sample of multi-ethnic people so that it can be used as a general survey of American teenagers. In addition, to our knowledge, our study is the largest research to investigate the link between adolescent serum vitamin D levels and VAT, which increases the study’s credibility.

## Conclusions

To sum up, this study found that serum vitamin D levels were inversely linked with VAT. This relationship depends on race and gender, and this negative relationship is only found in girls, as well as Mexican Americans and non-Hispanic whites. More prospective trials are necessary to further assess whether increasing serum vitamin D levels have a favorable effect on VAT in adolescents with vitamin D deficiency.

## Supplementary Information

**Additional file 1: Supplementary Table 1.** The missing data of covariates and their processing. **Supplementary Table 2.** Association between covariates and VAT mass. **Supplementary Table 3.** The adjusting roles of potential confounders on the estimates of serum vitamin D on VAT.

## Data Availability

Publicly available datasets were analyzed in this study. This data can be found here: https://wwwn.cdc.gov/nchs/nhanes/.

## Abbreviations

NHANES: National Health and Nutrition Examination Survey
VAT: Visceral adipose tissue
CVD: Cardiovascular disease
SAT: Subcutaneous adipose tissue
DXA: Dual-energy X-ray absorptiometry
BMI: Body mass index
