# Supplementary material for: Association between serum vitamin D levels and visceral adipose tissue among adolescents: a cross-sectional observational study in NHANES 2011–2015
Source: BMC Pediatr. 2022 Nov 4;22:634. doi: 10.1186/s12887-022-03688-2 (PMC9635166; doi:10.1186/s12887-022-03688-2)
Supplement: Supplementary file 1 — Additional file 1: Supplementary Table 1. The missing data of covariates and their processing. Supplementary Table 2. Association between covariates and VAT mass. Supplementary Table 3. The adjusting roles of potential confounders on the estimates of serum vitamin D on VAT. [file 12887_2022_3688_MOESM1_ESM.docx]

Supplementary table 1: The missing data of covariates and their processing.

| Covariant | complete data | Missing data | Process |
| --- | --- | --- | --- |
| Age | 3171 | 0 |  |
| Gender | 3171 | 0 |  |
| Race | 3171 | 0 |  |
| Ratio of family income to poverty | 2877 | 264 | Not recorded |
| Time of detection | 3171 | 0 |  |
| Sedentary activity | 3111 | 60 | Weighted mean |
| Waist circumference | 3127 | 24 | Weighted mean |
| BMI | 3141 | 30 | Not recorded |

BMI, body mass index.

Supplementary table 2: Association between covariates and VAT mass.

| covariates | Exp(beta),95%CI | P value |
| --- | --- | --- |
| Age | 9.09 (6.94, 11.25) | <0.0001 |
| Gender |  |  |
| Boys | 0 | 0 |
| Girls | -23.45 (-33.15, -13.76) | <0.0001 |
| Race |  |  |
| Mexican American | 0 | 0 |
| Other Hispanic | -39.70 (-61.22, -18.18) | 0.0003 |
| Non-Hispanic White | -41.06 (-55.07, -27.06) | <0.0001 |
| Non-Hispanic Black | -74.28 (-92.24, -56.32) | <0.0001 |
| Other Race | -41.88 (-62.38, -21.37) | <0.0001 |
| Ratio of family income to poverty |  |  |
| <1.3 | 0 | 0 |
| 1.3-1.8 | -0.26 (-17.58, 17.07) | 0.9767 |
| >1.8 | -28.85 (-39.96, -17.75) | <0.0001 |
| Not recorded | -9.38 (-30.67, 11.91) | 0.3878 |
| Time of detection |  |  |
| November 1 through April 30 | 0 | 0 |
| May 1 through October 31 | -19.14 (-28.86, -9.42) | 0.0001 |
| Sedentary activity | 0.03 (-0.00, 0.06) | 0.0846 |
| Waist circumference | 7.46 (7.28, 7.65) | <0.0001 |
| BMI |  |  |
| Underweight | 0 | 0 |
| Normal weight | 25.34 (6.23, 44.44) | 0.0094 |
| Overweight | 122.49 (102.08, 142.89) | <0.0001 |
| Obese | 265.23 (245.19, 285.27) | <0.0001 |
| Not recorded | 105.63 (64.61, 146.66) | <0.0001 |
| Serum vitamin D | -1.23 (-1.44, -1.02) | <0.0001 |
| Deficiency | 0 | 0 |
| Insufficiency | -23.42 (-34.94, -11.90) | <0.0001 |
| Sufficiency | -59.26 (-72.50, -46.02) | <0.0001 |

BMI, body mass index.

Supplementary table 3: The adjusting roles of potential confounders on the estimates of serum vitamin D on VAT.

| +/-covariates | Basic model | Complete model | Selected covariates |
| --- | --- | --- | --- |
| Age | -1.2407 | -0.3489 |  |
| Gender | -1.2110 | -0.3592 |  |
| Race | -1.6295 * | -0.1253 * | Yes |
| Ratio of family income to poverty | -1.1480 | -0.3672 |  |
| Time of detection | -1.1962 | -0.3565 |  |
| Sedentary activity | -1.2211 | -0.3457 |  |
| Waist circumference | -0.2697 * | -0.6627 * | Yes |
| BMI | -0.3497 * | -0.3627 | Yes |

*The change is more than 10% compared with the initial regression coefficient.
